# Jawbone mesenchymal stromal cells attenuate acute inflammation via hematopoietic niche reinforcement

**DOI:** 10.3389/fbioe.2025.1596143

**Published:** 2025-08-11

**Authors:** Xinyu Wang, Qianye Chen, Jiping Sun, Zihan Huang, Zijian Zhang, Tingwei Lu, Xiangru Huang, Siyuan Sun, Yuanqi Liu, Houwen Pan, Li Mei, Paul R. Cooper, Qinggang Dai, Lei Shen, Lingyong Jiang

**Affiliations:** ^1^ Center of Craniofacial Orthodontics, Shanghai Ninth People’s Hospital, Shanghai Jiao Tong University School of Medicine, Shanghai Research Institute of Stomatology, Shanghai, China; ^2^ Department of Oral and Maxillofacial-Head and Neck Oncology, Shanghai Ninth People’s Hospital, Shanghai Jiao Tong University School of Medicine, Shanghai, China; ^3^ Center for Immune-Related Diseases at Shanghai Institute of Immunology, Ruijin Hospital, Shanghai Jiao Tong University School of Medicine, Shanghai, China; ^4^ Faculty of Dentistry, Sir John Walsh Research Institute, University of Otago, Dunedin, New Zealand; ^5^ Shanghai Key Laboratory of Stomatology, The 2nd Dental Center, Shanghai Ninth People’s Hospital, Shanghai Jiao Tong University School of Medicine, Shanghai Research Institute of Stomatology, Shanghai, China

**Keywords:** jawbone mesenchymal stem cells, hematopoietic stem cells, acute infection, exocrine, cell therapy

## Abstract

**Background:**

The bone marrow microenvironment, comprising various cell types and molecular signals, finely orchestrates the self‐renewal and lineage commitment of hematopoietic stem cells (HSCs). Although most investigations have centered on mesenchymal stem cells (MSCs) from long bones, the distinct properties and immunoregulatory functions of craniofacial bone marrow derived MSCs remain largely unexplored. Notably, jawbone MSCs not only exhibit a robust capacity for promoting hematopoietic regeneration but also offer therapeutic potential in infectious diseases.

**Methods:**

Using an optimized enzymatic digestion protocol, we obtained a highly viable single‐cell suspension from mouse jawbone *in vitro*. Single‐cell sequencing was then performed to explore the interactions between jawbone MSCs and HSCs, while tissue immunofluorescence clarified their spatial distribution. *In vitro* osteogenic and adipogenic differentiation assays confirmed the multilineage potential of jawbone MSCs. A biomimetic co‐culture system, designed to emulate the bone marrow niche, was employed to assess the impact of jawbone MSCs on HSC differentiation, which was evaluated via flow cytometry. Mechanistic insights into HSC changes were gleaned from RT‐qPCR and cellular immunofluorescence. Subsequently, an LPS‐induced acute infection model was established to evaluate the therapeutic efficacy of jawbone MSCs. Finally, comprehensive analysis of single‐cell sequencing data, in conjunction with RT‐qPCR findings, elucidated the regulatory pathways through which jawbone MSCs promote hematopoiesis.

**Results:**

Single‐cell sequencing revealed a robust interaction between jawbone MSCs and HSCs. Tissue immunofluorescence demonstrated that in the mouse jawbone, MSCs and HSCs were located in close spatial proximity. *In vitro* osteogenic and adipogenic induction experiments showed that jawbone MSCs possess considerable multilineage differentiation potential. Co‐culture assays further indicated that jawbone MSCs induce HSCs to differentiate into various immune cell types, particularly promoting B cell generation. RT‐qPCR and immunofluorescence assays confirmed that pivotal transcription factors, such as PAX5, were activated in B cells. In an *in vivo* infection model, jawbone MSCs exhibited significant anti‐infective capabilities, effectively reducing mortality and systemic inflammation in infected mice. A deeper analysis of the single‐cell sequencing data revealed that jawbone MSCs mainly facilitate hematopoiesis by secreting CXCL12.

**Conclusion:**

Through single‐cell sequencing, *in vitro* multilineage induction, co‐culture systems, and a mouse model of LPS‐induced acute infection, this study systematically elucidates the close interplay between jawbone MSCs and HSCs, as well as their pivotal roles in immune modulation and anti‐infective responses. The findings demonstrate that jawbone MSCs not only exhibit robust multilineage differentiation potential but also secrete CXCL12 and activate key B cell transcription factors (such as PAX5). This process significantly promotes HSC differentiation into B cells, improves survival rates in infected mice, and attenuates systemic inflammation. These results establish a strong foundation for further investigation into the applications of jawbone MSCs in immune regulation and disease therapy.

## 1 Introduction

The bone marrow microenvironment, comprising various cell types and molecular signals, precisely orchestrates the self‐renewal and lineage commitment of hematopoietic stem cells (Pinho and Frenette, 2019). While the marrow in long bones plays a pivotal role in hematopoiesis and immune homeostasis, it undergoes degenerative structural and functional changes with advancing age (de Haan and Lazare, 2018). Aging disrupts the marrow niche in long bones, characterized by red marrow conversion into yellow marrow, an increased number of adipocytes, and declining hematopoietic stem cell functionality (Ross et al., 2024). These cumulative impairments reduce the regenerative potential of hematopoietic progenitors, eventually compromising effective immune responses and heightening susceptibility to infections, tumors, and other illnesses (Nakamura-Ishizu et al., 2020). Recent evidence indicates that not all regions of the bone marrow exhibit uniform senescent deterioration. Compared with long bone marrow, the craniofacial bone marrow niche displays superior resistance to aging. Jawbone marrow maintains a high proportion of hematopoietic progenitors throughout life, retaining robust self‐renewal and differentiation capacities (Koh et al., 2024). These observations underscore the unique strengths of jawbone marrow in sustaining hematopoietic function, modulating immune responses, and conferring anti‐aging benefits, thereby broadening the theoretical framework of the bone–immune axis.

Notably, craniofacial bones differ significantly from long bones in their physiological properties, architecture, and functions (Aghaloo et al., 2010). During embryonic development, jaw osteoblasts primarily derive from neural crest cells, whereas those in long bones originate mainly from the mesoderm—leading to distinctions in developmental pathways, functional regulation, and responsiveness to external stimuli (Yang et al., 2024). Moreover, jaw osteoblasts play specialized roles in sustaining and remodeling the alveolar bone microenvironment (Fleischmannova et al., 2010). Beyond bone formation, they interact with root fibrous tissues and the periodontal ligament to ensure tooth stability. These differences in embryonic origin, differentiation control, and functionality imply that jawbone mesenchymal stem cells may possess distinctive physiological features.

Hematopoietic stem cells, which exhibit self‐renewal and multilineage differentiation potential, generate multiple immune cell types (Eibel et al., 2014). Primarily located in the bone marrow, hematopoietic stem cells support B cell differentiation and maturation. Immature B cells undergo gene rearrangement in the marrow, expressing functional B cell receptors and advancing through various B‐cell precursor stages (Zhong et al., 2023), before migrating to peripheral lymphoid organs (e.g., spleen and lymph nodes) to complete maturation and execute immune functions (Doores, 2023). Their differentiation is regulated by diverse biological signals, including antigens, cytokines, and helper T cells (Kloc et al., 2024). Due to the jawbone’s distinctive marrow cavity and chronic exposure to oral microorganisms, it hosts a specialized immune microenvironment (Cekici et al., 2000). Within the jawbone, B cells produce immunoglobulins (IgA, IgG, IgM) to combat oral pathogens, bolstering antibody‐mediated immunity during periodontitis (Zou et al., 2022; Zhang et al., 2024). Under conditions of jawbone or odontogenic infection, B cells also secrete the anti‐inflammatory cytokine IL‐10, limiting excessive immune responses and promoting bone repair and regeneration (Shi et al., 2020; Liu et al., 2024). However, whether direct interactions exist between jaw osteoblasts and B cells, as well as the precise underlying mechanisms, remains unclear.

In this study, we systematically mapped the spatial relationships between mesenchymal stem cells and hematopoietic stem cells within mouse jawbone tissue. Using *in vitro* co‐culture systems, we explored how jawbone mesenchymal stem cells regulate hematopoietic stem cell differentiation and development and investigated the specific mechanisms involved. Moreover, by establishing a mouse model of acute infection, we demonstrated the remarkable immunotherapeutic efficacy of jawbone mesenchymal stem cells. These findings offer valuable insights for immune regulation and therapeutic strategies in infectious diseases.

## 2 Materials and methods

### 2.1 Animals

Col1-CreERT2 transgenic mice (C57BL/6 background) were genetically crossed with ROSA26-loxP-tdTomato reporter strains to establish a dual-recombinant lineage (Col1-CreERT2; tdTomato+). Male specimens at 6 weeks postnatal development were selected for all experimental cohorts. The parental Col1-CreERT2 strain and ROSA26Sortm14 (CAG-tdTomato) Hze/J line were obtained through academic collaboration with Dr. Bin Zhou’s laboratory at the Shanghai Institutes for Biological Sciences, Chinese Academy of Sciences. This combinatorial genetic strategy enables precise lineage tracing of collagen type I-expressing mesenchymal progenitors and their cellular derivatives through tamoxifen-inducible fluorescent labeling. Experimental animals were maintained in pathogen-free facilities with controlled environmental parameters (ambient temperature 22°C ± 2°C, relative humidity 52% ± 3%), receiving autoclaved rodent chow and sterile hydration *ad libitum*. All investigative procedures received institutional review board approval (Ethics Committee of Ninth People’s Hospital Affiliated to Shanghai Jiao Tong University School of Medicine.

### 2.2 Isolation and culture of mouse jawbone mesenchymal stem cells

Following cervical dislocation, the mice were immersed in 75% ethanol for 15 min. After removing the facial skin, both mandibles were dissected and placed in PBS. Periosteum and adjoining connective tissues were carefully stripped away, and the mandibles were rinsed 2–3 times with PBS until all traces of blood were cleared. Bone tissues were first minced into approximately 1 mm^3^ fragments using sterile ophthalmic scissors, followed by digestion with collagenase I. An appropriate amount of 0.1% collagenase type I was added, and the tissue was digested for 10 min; the supernatant was again discarded. Subsequently, four rounds of digestion were carried out with 0.1% collagenase type I for 20 min each at 37°C. The supernatants collected from these four steps were transferred to a centrifuge tube, and 5 mL of α-MEM containing 10% FBS was added to halt digestion. The combined solution was passed through a 150-mesh filter three times, then centrifuged at 300 ×g for 10 min, after which the supernatant was discarded. The cell pellet was resuspended in expansion medium (α-MEM supplemented with 10% FBS [HyClone], 1% penicillin/streptomycin [Gibco]), thoroughly mixed, and counted. Cells were cultured at 37°C in a 5% CO_2_ incubator. The medium was replaced every 3 days until 80%–90% confluence was achieved, after which cells were subcultured with 0.25% trypsin-EDTA (Gibco). Cells at passages 3–5 (P3–P5) were utilized for all downstream experiments.

### 2.3 Multilineage differentiation assays of cells

For osteogenic differentiation, cells were seeded at a density of 2 × 10^5^ cells/mL in osteogenic induction medium (OriCell) and cultured for 21 days. For adipogenic differentiation, cells were seeded at 1.5 × 10^5^ cells/mL in adipogenic induction medium (OriCell).

### 2.4 Adipogenic differentiation staining

Lipid droplets were stained with Oil Red O (Oricell). Briefly, cells were fixed with 4% paraformaldehyde, incubated with Oil Red O working solution for 10 min, washed with PBS, and imaged.

### 2.5 Alkaline phosphatase staining of cells

Cultured osteoprogenitor cells underwent 7-day osteogenic differentiation induction. Following medium aspiration, specimens were subjected to triple PBS washing cycles and 15-min fixation in 4% paraformaldehyde (PFA) at ambient conditions (22°C ± 1°C). Cellular alkaline phosphatase activity was visualized using BCIP/NBT chromogenic substrates (Beyotime, China) per manufacturer’s protocol. Substrate incubation proceeded in light-protected environments at 37°C until distinct violet chromogenic products emerged (15–30 min observation window). Termination was achieved through triple deionized water rinses, with staining intensity quantitatively reflecting enzymatic activity levels through spectrophotometric analysis.

### 2.6 Alizarin red staining of cells

Three-week osteogenic culture with biweekly medium replenishment facilitated extracellular matrix mineralization. Post-culture processing included PBS washing sequences and 10-min PFA fixation at standard laboratory conditions. Mineralized nodules were identified through 20-min Alizarin Red S (pH 4.2, Beyotime, China) immersion at 24°C, followed by destaining with calcium-free aqueous solutions until negative controls demonstrated optical clarity. Quantitative assessment of calcium accumulation was performed through acetic acid extraction and spectrophotometric measurement at 405 nm, with positive mineralization evidenced by characteristic crimson staining patterns under phase-contrast microscopy.

### 2.7 Lin−Sca1+c-kit (LSK) cells sorting

After euthanizing the mice, the femurs and tibias were isolated using ophthalmic scissors. In brief, all surrounding muscles were removed, and a syringe filled with PBS containing 2% FBS was used to flush the bone marrow cavities. A cell suspension was generated by grinding the cells through a needle. The cells were treated with red blood cell lysis buffer for 5 min to lyse all red blood cells, followed by washing with PBS. The cells were then stained for 30 min with anti-mouse lineage cocktail, Sca1, and c-Kit antibodies. Finally, the LSK cells were sorted using flow cytometry.

### 2.8 Co-culture assay

Added content: A Transwell system (0.4 μm pore size, Corning) was used. MSCs of different concentrations (5 × 10^4^, 2.5 × 10^5^, and 5 × 10^5^ cells per well) were seeded in the upper chamber, while LSK cells (5 × 10^4^) cells per well were placed in the lower chamber. After 7 days of co-culture, cells from lower chambers were collected for analysis.

### 2.9 Flow cytometry

After collecting the cells, they were centrifuged (300 g, 4°C, 5 min) and resuspended in flow cytometry buffer, with the cell concentration adjusted to 1-2 × 10^6^ cells/mL. An appropriate volume of the cell suspension (∼100 µL per tube) was aliquoted into flow cytometry tubes. Fluorescently labeled antibodies (targeting CD3, CD4, CD8, CD19, B220, CD11b, Gr-1, etc.) and a viability dye (such as 7-AAD or PI) were added to each tube, following the manufacturer’s recommended antibody concentrations, and gently mixed. The cell mixtures were incubated at 4°C in the dark for 30 min. After incubation, 1 mL of flow cytometry buffer was added to wash the cells, followed by centrifugation at 300 g, 4°C, for 5 min. The supernatant was aspirated, and the cells were resuspended in 500 µL of flow cytometry buffer, ready for flow cytometric analysis. Flow cytometry was performed to analyze the stained cells, with appropriate lasers and filters set to detect each fluorescent marker. After excluding dead cell signals, fluorescence signals from T cells (CD3, CD4, CD8), B cells (CD19, B220), and myeloid cells (CD11b, Gr-1) were sequentially detected.

### 2.10 RT-qPCR

RNA was extracted from the cells using a kit (Vazyme, FastPure Cell/Tissue Total RNA Isolation Kit) according to the manufacturer’s instructions. RNA was then reverse-transcribed into cDNA using the Takara reverse transcription kit. PCR was performed using SYBR Green PreMix (Tiangen) on a LightCycler 96 system (Roche, Basel, Switzerland) to quantify mRNA levels. The 2^−ΔΔCT^ method was used to analyze the RT-PCR data. The primer sequences used in this study are listed in Supplementary Table S1.

### 2.11 Preparation of frozen sections of alveolar tissue

Following euthanasia, the oral cavity was opened with scissors and forceps to expose the alveolar region. Soft tissues around the jawbone were removed with care to prevent damage to the alveolar bone and teeth. The alveolar tissue was excised in a single piece and immediately placed in PBS to clear any residual blood. It was then transferred into 4% PFA at 4°C for 24 h. After fixation, the sample was immersed in 30% sucrose at 4°C overnight (usually 12–24 h) until it sank, indicating complete dehydration. Excess solution was gently blotted away, and the tissue was positioned in a mold filled with optimal cutting temperature (OCT) compound so that it would be correctly oriented for sectioning. The mold was flash‐frozen on dry ice or in liquid nitrogen and stored at −80°C once fully solidified. For cryosectioning, the frozen block was moved from −80°C to a cryostat (approximately −20°C) and allowed to equilibrate for 10–20 min. The tissue was mounted on the cryostat stage and sectioned at a thickness of 10–12 µm. Each section was carefully placed on a chilled slide, ensuring it lay flat and remained intact. Slides can be kept at −20°C for short‐term storage or at −80°C for longer durations.

### 2.12 Immunofluorescence staining

Frozen sections were taken from −20°C or −80°C storage and briefly allowed to air-dry at room temperature (RT) for 10 min. They were then fixed with 4% paraformaldehyde (PFA) at 4°C for 10–15 min. After three 5-min washes in PBS, sections were permeabilized with 0.1% Triton X-100 (in PBS) for 10–15 min and washed again three times, 5 min each. Next, a blocking solution (5% BSA or 10% normal goat serum) was applied in a humid chamber at 37°C for 30 min to minimize nonspecific binding. The blocking solution was removed, and diluted primary antibodies were added to cover the sections. Incubations took place overnight at 4°C or for 1–2 h at 37°C. Following another trio of 5-min washes in PBS, a fluorescently labeled secondary antibody was applied and protected from light for 1 h at 37°C or 1–2 h at 4°C. After three more 5-min washes in PBS, DAPI solution (1 μg/mL) was added for 5–10 min at RT in the dark to stain nuclei, then washed off in three additional 5-min PBS rinses. Finally, antifade mounting medium was placed on each section, and a coverslip was carefully lowered to avoid bubbles and ensure complete coverage. The prepared sections were examined under a fluorescence microscope using appropriate channels for both the target antigen and DAPI.

### 2.13 Establishment of mouse acute infection model

Male C57BL/6 mice (6–8 weeks old) were randomly assigned to groups and labeled. They fasted for 12 h prior to experimentation but had free access to water. An LPS solution at 5 mg/kg was prepared according to each mouse’s body weight, then diluted with normal saline. Intraperitoneal injection was performed using a syringe positioned slightly to the side of the lower abdominal midline; the injection volume was minimized to reduce the risk of organ damage. Afterward, mice were placed in a warm setting and closely monitored for body temperature, weight, activity, and overall condition, with prompt intervention for any abnormalities. All mice were euthanized at 7 days post-infection through intraperitoneal administration of 200 mg/kg sodium pentobarbital. Unconsciousness was verified by absent pedal reflex prior to subsequent procedures, consistent with institutional ethical guidelines.

### 2.14 ELISA

Peripheral serum was collected from mice, and the cell-free supernatants were stored at −80°C until analysis. Samples were diluted with PBS at the specified ratio and the concentrations of IL-6 and IFN-γ were measured using commercially available ELISA kits (Biolegend) according to the manufacturer’s instructions. During the assay, a microplate reader (BioTek Epoch) was used to measure the optical density (OD) of both standards and samples, and cytokine concentrations were calculated based on the standard curve to ensure data accuracy and reproducibility.

### 2.15 Statistical analyses

All statistical assessments were carried out using GraphPad Prism 9, employing Student’s t‐test or ANOVA to determine significance. A p‐value <0.05 (*p < 0.05, **p < 0.01, ***p < 0.001, ****p < 0.0001) was considered statistically significant.

## 3 Results

### 3.1 Single-cell sequencing-based analysis of the relationship between mesenchymal stem cells and hematopoietic stem cells in the jawbone

**FIGURE 1:**
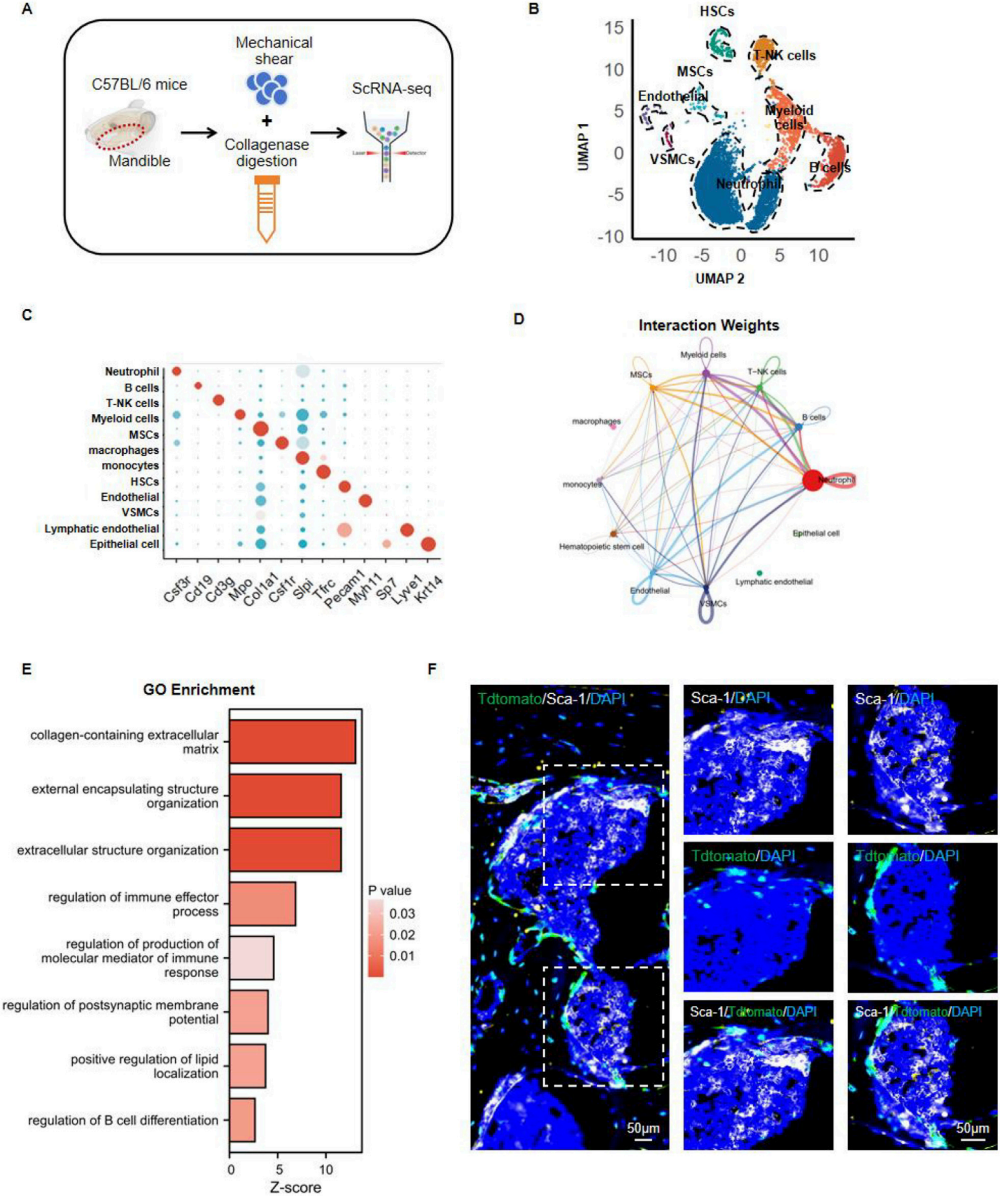
Analyzing the interaction between mesenchymal stem cells and hematopoietic stem cells in the jawbone. **(A)** Mandibles from 6‐weeks‐old C57BL/6 mice were processed using mechanical shear and collagenase digestion to create single‐cell suspensions, followed by high‐throughput single‐cell RNA sequencing (ScRNA‐seq). **(B)** UMAP‐based dimensionality reduction clusters the cells into distinct groups. **(C)** Bubble plot illustrating the expression of characteristic genes across different clusters. **(D)** Intercellular communication network showing ligand–receptor interaction weights. **(E)** Gene Ontology (GO) enrichment bar chart depicting key biological pathways. **(F)** Representative multiplex immunofluorescence images highlighting Sca‐1 (green), TdTomato (red), and DAPI (blue) signals within mandible tissue.

In order to investigate how mesenchymal stem cells (MSCs) and hematopoietic stem cells (HSCs) interact within mouse jawbone tissue, we isolated mandibles from six-week-old adult mice and performed single‐cell sequencing. Owing to the inherent hardness of jawbone, its high level of extracellular matrix mineralization, and the tightly connected cellular architecture, conventional enzymatic digestion methods often fail to produce single‐cell suspensions from this tissue. Drawing on earlier publications, we adopted an optimized protocol that combines enzymatic digestion with mechanical disruption (Jin et al., 2023). Specifically, collagenase and trypsin digestion were paired with gentle mechanical grinding, enabling us to maximize cell viability and increase the yield of single cells. By applying this approach, we successfully obtained highly viable single‐cell suspensions, which were then subjected to high‐throughput single‐cell transcriptome sequencing on the 10x Genomics platform to further dissect both the heterogeneity and functional attributes of jawbone cells (Figure 1A). We utilized UMAP (Uniform Manifold Approximation and Projection) for dimensional reduction and visualization of the resulting single‐cell data, categorizing the cells into eight distinct clusters based on their gene expression patterns (Figure 1B). A bubble plot illustrated the expression of various genes across these clusters, with bubble size and color signifying gene expression levels; changes in size and color reflect the overall expression strength and abundance for each gene in different cell populations (Figure 1C). To investigate interactions among these cell groups, we employed CellChat to analyze the inter‐cluster signaling. A network diagram was used to depict interaction weights, representing communication strength between different cell populations. Each node indicates a particular cell cluster, while the thickness and color of connecting lines convey the magnitude of their interactions. Notably, MSCs exhibited strong associations with several immune cell types, most prominently B cells and neutrophils (Figure 1D). Gene Ontology (GO) enrichment analysis of MSCs showed that key biological pathways such as extracellular matrix organization, immune effector processes, and immune response modulation were significantly enriched (Figure 1E). In addition, we conducted multiplex immunofluorescence staining to characterize the spatial distribution of MSCs and HSCs in the mouse mandible. We employed fluorescent tracer mice to label Col1^+^ mesenchymal stem cells and their progeny. The fluorescence signals of Tdtomato^+^ mesenchymal stem cells were found to be closely localized to those of Sca1^+^ hematopoietic stem cells (Figure 1F). Taken together, these findings suggest that in the mouse jawbone microenvironment, there is a potential interactive relationship between bone marrow‐derived mesenchymal stem cells and hematopoietic stem cells.

### 3.2 Assessment of the multilineage differentiation potential of jawbone mesenchymal stem cells

**FIGURE 2:**
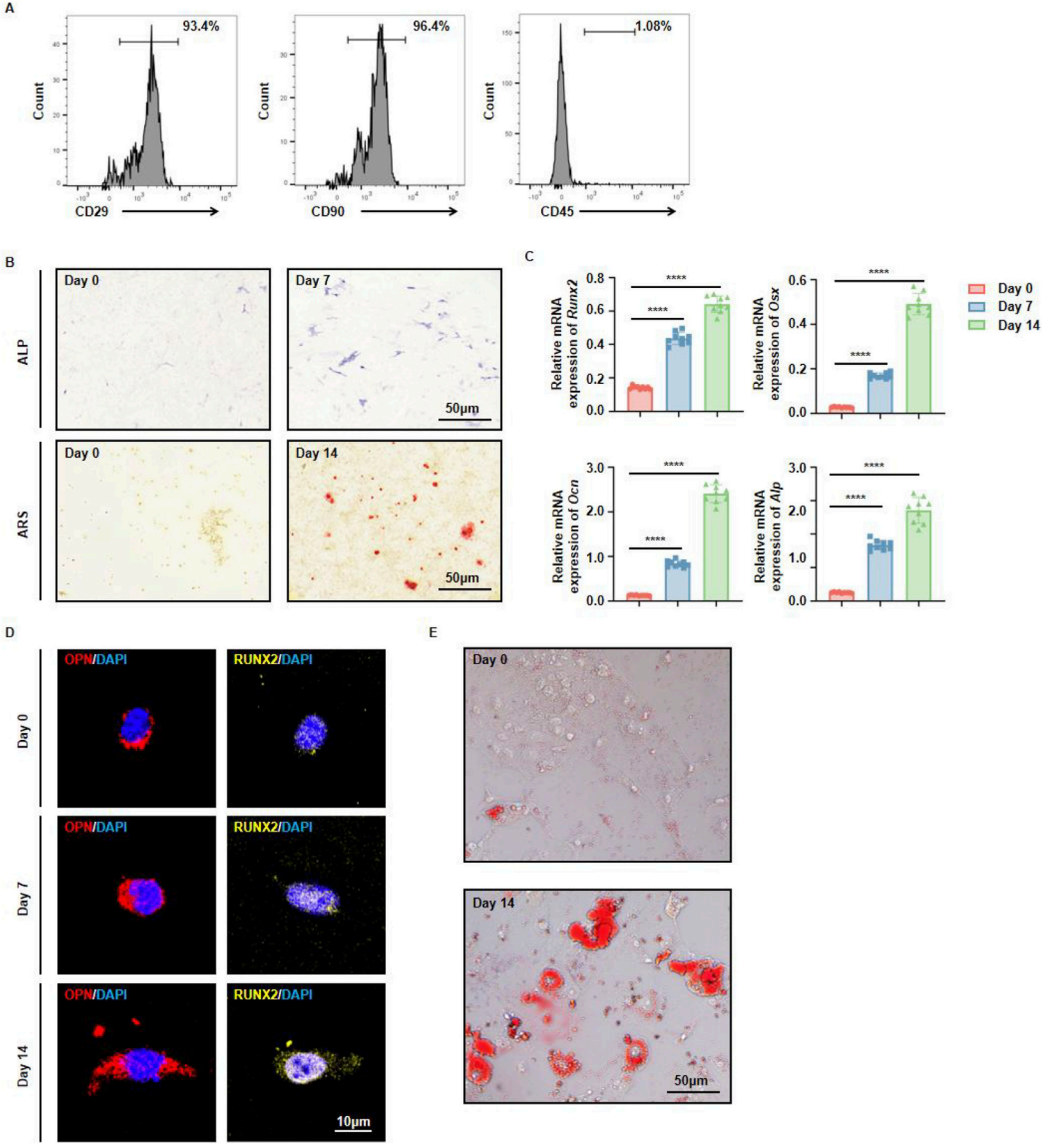
Multidirectional differentiation potential of jawbone mesenchymal stem cells. **(A)** Flow cytometric analysis of cell surface markers CD29, CD90, and CD45. **(B)** Alkaline phosphatase (ALP) staining on day 0 vs. day 7 (upper panels) and Alizarin Red S (ARS) staining on day 0 vs. day 14 (lower panels) reveal progressive osteogenic maturation over time. Scale bar, 50 µm. **(C)** Relative mRNA levels of the osteogenic markers Runx2, Osx, Ocn, and Alp at days 0, 7, and 14. Bars represent the mean ± standard error; statistical significance is indicated. **(D)** Immunofluorescent detection of osteopontin (OPN, red) and RUNX2 (yellow), with nuclei counterstained by DAPI (blue), shows increased signal intensity on days 7 and 14. Scale bar, 10 µm. **(E)** *In vitro* adipogenic induction and differentiation of jawbone mesenchymal stem cells. Scale bar, 50 µm.

MSCs are a type of adult stem cell with multipotent differentiation capacities, enabling them to give rise to osteoblasts, chondrocytes, and adipocytes. While the osteogenic and adipogenic potentials of MSCs from long bones have been extensively investigated, there has been comparatively little systematic evaluation of the *in vitro* differentiation abilities of mouse jawbone MSCs. In light of the jawbone’s critical role in oral repair and regenerative medicine, we explored the *in vitro* differentiation potential of mouse jawbone MSCs. Using the enzymatic digestion method described previously, we obtained a single‐cell suspension from the jawbone. After 7 days of culture, the adherent cells were identified as jawbone MSCs. Flow cytometry analysis revealed that these adherent cells strongly expressed the typical MSC markers CD29 and CD90, while being negative for the hematopoietic marker CD45 (Figure 2A). Following 7 days of osteogenic induction, alkaline phosphatase (ALP) staining indicated elevated ALP secretion. By day 14 of osteogenic induction, the jawbone MSCs had formed distinct mineralized calcium nodules (Figure 2B). RT‐qPCR analyses showed that compared with the non‐induced control group, the expression levels of osteogenesis‐associated genes (Runx2, Osx, Ocn, and Alp) were significantly upregulated and positively correlated with the induction period (Figure 2C). Immunofluorescence staining further demonstrated that osteogenic markers OPN and RUNX2 increased in expression in a time‐dependent manner (Figure 2D). When these cultured MSCs underwent adipogenic induction, they likewise exhibited robust adipogenic potential relative to their non‐induced counterparts (Figure 2E). Taken together, these findings indicate that by employing an optimized enzymatic digestion protocol, we successfully isolated and expanded jawbone MSCs *in vitro*, which display strong multipotent differentiation capacities.

### 3.3 Constructing an *in vitro* biomimetic bone marrow microenvironment to validate the regulatory effects of jawbone mesenchymal stem cells on hematopoietic stem cells

**FIGURE 3:**
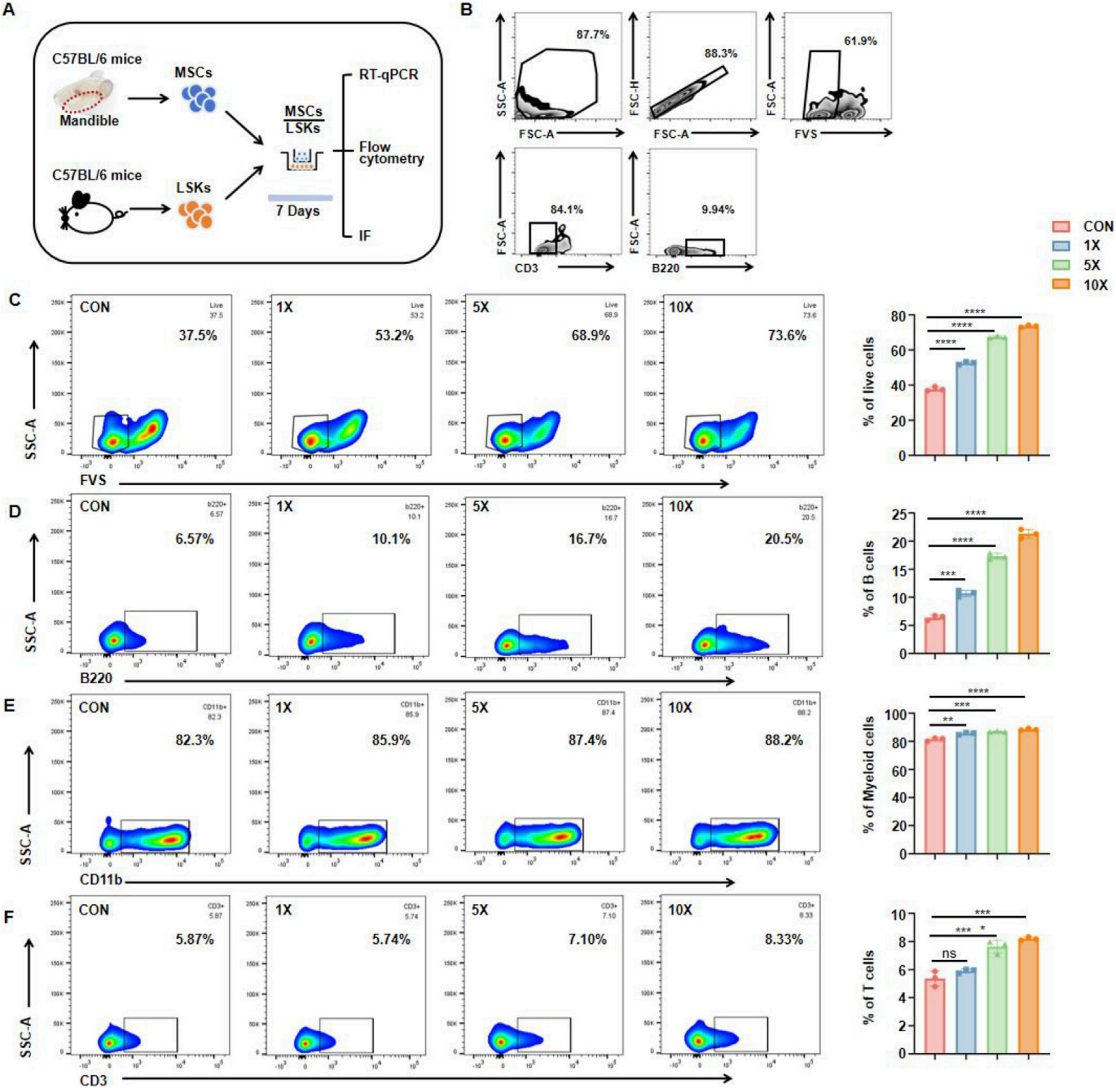
Mesenchymal stem cells from jawbone induce differentiation of hematopoietic stem cells into different types of immune cells *in vitro*. **(A)** Experimental schematic, IF immunofluorescence. **(B)** Representative gating strategy. **(C)** Flow cytometry plots (left) and quantification (right) showing live cell percentages (FVS^−^) under increasing MSC concentrations. **(D)** Proportions of B220^+^ B cells rise in a dose‐dependent manner with higher MSC concentrations, illustrating the MSCs’ potential to drive B cell development. **(E)** CD11b^+^ myeloid cells also display a mild yet statistically significant increase, indicative of broader hematopoietic support by jawbone MSCs. **(F)** CD3^+^ T cells exhibit a modest but notable uptick under co‐culture with higher MSC concentrations. Bar graphs represent mean ± SEM, and asterisks denote levels of statistical significance. Statistical analysis of the ratio and quantity of live cells and B cells. n = 3 for each group (*p < 0.05, **p < 0.01, ***p < 0.001 and ****p < 0.0001). “CON” refers to the group without MSCs addition, “×1,” “×5,” and “×10” indicate MSC-to-target cell co-culture ratios (1:1, 5:1, 10:1).

A Transwell co‐culture system was established under optimized conditions to simulate a biomimetic bone marrow environment. MSCs were cultured in the upper chamber to establish the experimental system for studying intercellular communication. A Transwell insert with 0.4‐µm pores permitted the exchange of soluble proteins and other factors while preventing direct cell–cell contact. Additionally, the entire system was maintained in a low‐oxygen environment (5% O_2_) to approximate the hypoxic niche found in actual bone marrow. By refining the seeding densities of MSCs and HSCs and adjusting co‐culture duration, we further replicated the densely packed, dynamic interactions characteristic of bone marrow. The use of specialized stem cell culture medium provided biochemical signals analogous to those of the endogenous marrow microenvironment, resulting in a reliable *in vitro* platform for examining how MSC‐secreted factors influence hematopoiesis (Figure 3A). Different quantities of jawbone MSCs (5 × 10^4^, 2.5 × 10^5^, and 5 × 10^5^ cells per well) were placed in the upper chamber to establish an osteogenic cell density gradient, while LSK cells (5 × 10^4^ cells per well) were cultured in the lower chamber. Before co‐culture, a standard gating strategy was used to remove nonviable cells, ensuring that only the targeted cell population proceeded to the following analyses. After 7 days, immune cells in the lower chamber were collected and assessed. Flow cytometry, also employing a standard gating procedure, was performed to track the differentiation and maturation of three classic immune cell populations: CD3^+^ T cells, CD11b^+^ myeloid cells, and B220^+^ B cells (Figure 3B). Results indicated that jawbone MSCs increased overall immune cell activity in a concentration‐dependent manner (Figure 3C) and likewise promoted the formation of diverse immune cell types (Figures 3D–F). Notably, MSCs elevated the proportion of B cells by nearly threefold (Figure 3D). Taken together, these findings demonstrate that jawbone MSCs effectively enhance the generation of downstream immune cells derived from hematopoietic stem cells, particularly facilitating the development and survival of B cells.

### 3.4 Jawbone mesenchymal stem cells promote the development of hematopoietic stem cells into B cells via Pax5

**FIGURE 4:**
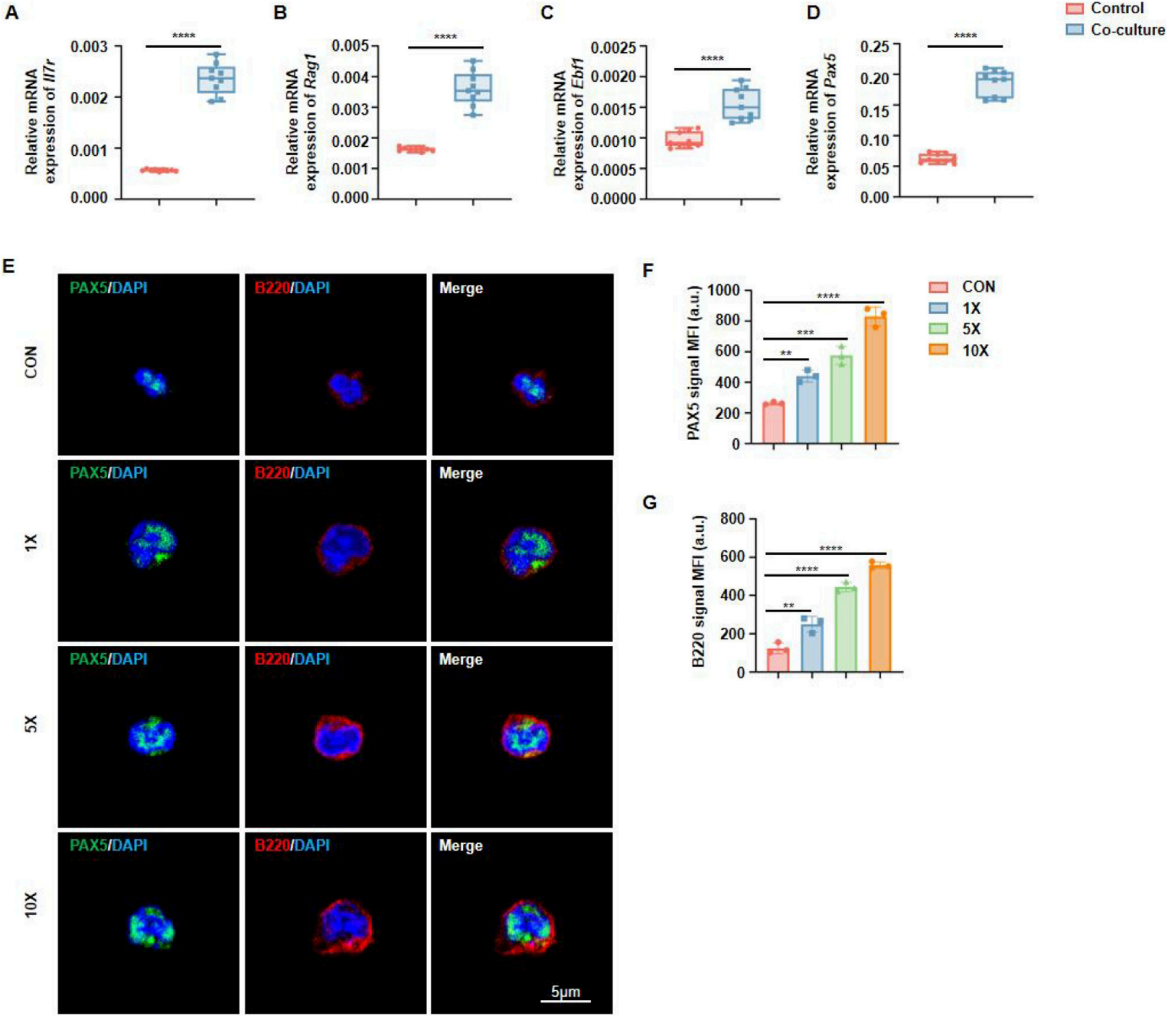
Jawbone mesenchymal stem cells promote hematopoietic stem cell generation through PAX5. **(A–D)** RT-qPCR is used to detect key transcription genes during B cell development. n = 9 for each group. **(E)** Immunofluorescence detection of the expression abundance of Pax5 and B220 in immune cells. **(F, G)** Calculation and analysis of the Mean Fluorescence Intensity (MFI). n = 3 for each group (*p < 0.05, **p < 0.01, ***p < 0.001 and ****p < 0.0001). “CON” refers to the group without MSCs addition, “×1,” “×5,” and “×10” indicate MSC-to-target cell co-culture ratios (1:1, 5:1, 10:1).

EBF1, RAG1, IL7R, and Pax5 are critical transcription factors involved in B cell development, playing indispensable roles in immunoglobulin gene rearrangement, B cell receptor expression, and B cell maturation. Using RT-qPCR, we compared the expression of key regulatory genes under co-culture and monoculture conditions. The results showed that, compared to HSCs cultured alone, those stimulated by MSCs exhibited elevated expression of Ebf1, Rag1, Il7r, and Pax5 (Figures 4A–D). Among these, Pax5 functions as a central regulator of B cell differentiation and maturation. Immunofluorescence staining revealed that MSCs significantly upregulated Pax5 expression in hematopoietic lineage precursor cells, indicating their potential role in promoting B cell differentiation (Figure 4E). Quantitative fluorescence analysis revealed a pronounced positive correlation between MSCs density and the expression of PAX5 and B220 (Figures 4F–G).

### 3.5 Jawbone mesenchymal stem cells can be used to treat acute infections

**FIGURE 5:**
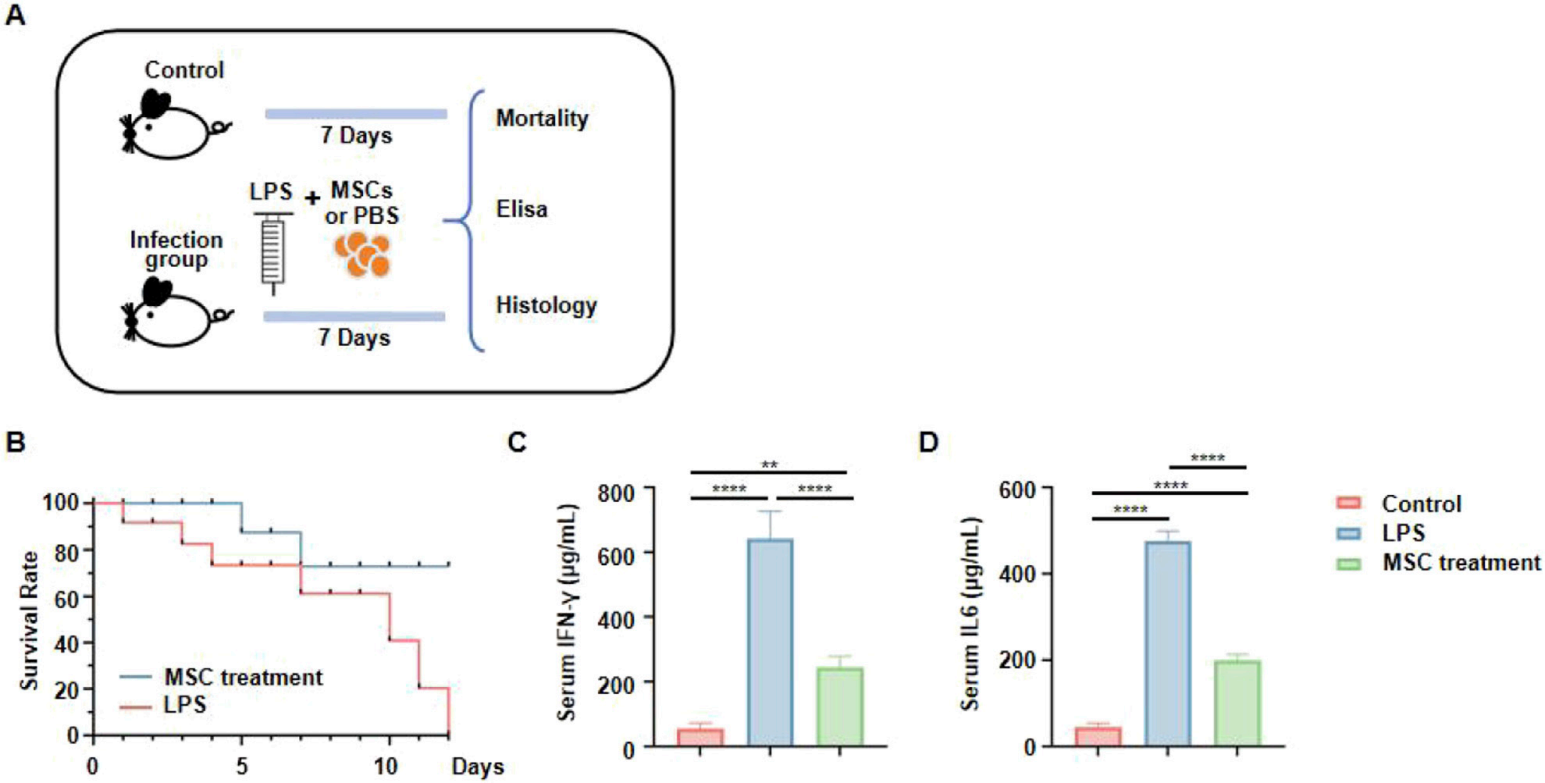
Jawbone mesenchymal stem cells can be used to treat acute infections. **(A)** Experimental schematic. Mice were divided into a control group (no LPS), and an infection group that received LPS injections. Animals in the infection group were then treated with either mesenchymal stem cells (MSCs) or PBS for 7 days. Survival rates and serum cytokine levels were assessed. **(B)** Survival curves. The MSC-treated group (blue line) exhibits a markedly higher survival rate compared to the untreated LPS group (red line). **(C, D)** Serum IFN-γ and IL6 concentrations, measured via ELISA.

To further examine the therapeutic effects of jawbone MSCs in vivo, we employed a mouse model of lipopolysaccharide (LPS)–induced infection. Mice were divided into a control group and an infection group; the latter received LPS injections to elicit inflammation. Following this, infected mice were treated with MSCs or PBS (placebo) and monitored over a seven-day period. At the end of the study, survival rates and serum cytokine levels were evaluated (Figure 5A). Monitoring mortality revealed that the LPS group experienced a high death rate, whereas the MSC-treated group demonstrated markedly improved survival, indicating that MSCs confer protection against LPS-induced lethality (Figure 5B). Further evaluation of systemic inflammation was conducted by measuring serum IFN-γ and IL-6 levels. These analyses showed a marked increase in inflammatory cytokines in LPS-infected mice, which was significantly reduced by MSC treatment, suggesting a notable anti-inflammatory effect of jawbone MSCs (Figures 5C–D). These findings demonstrate that in an acute LPS-induced infection model, jawbone MSC therapy not only improves survival rates but also lowers levels of inflammatory cytokines.

### 3.6 Jawbone mesenchymal stem cells produce CXCL12 to regulate hematopoiesis

**FIGURE 6:**
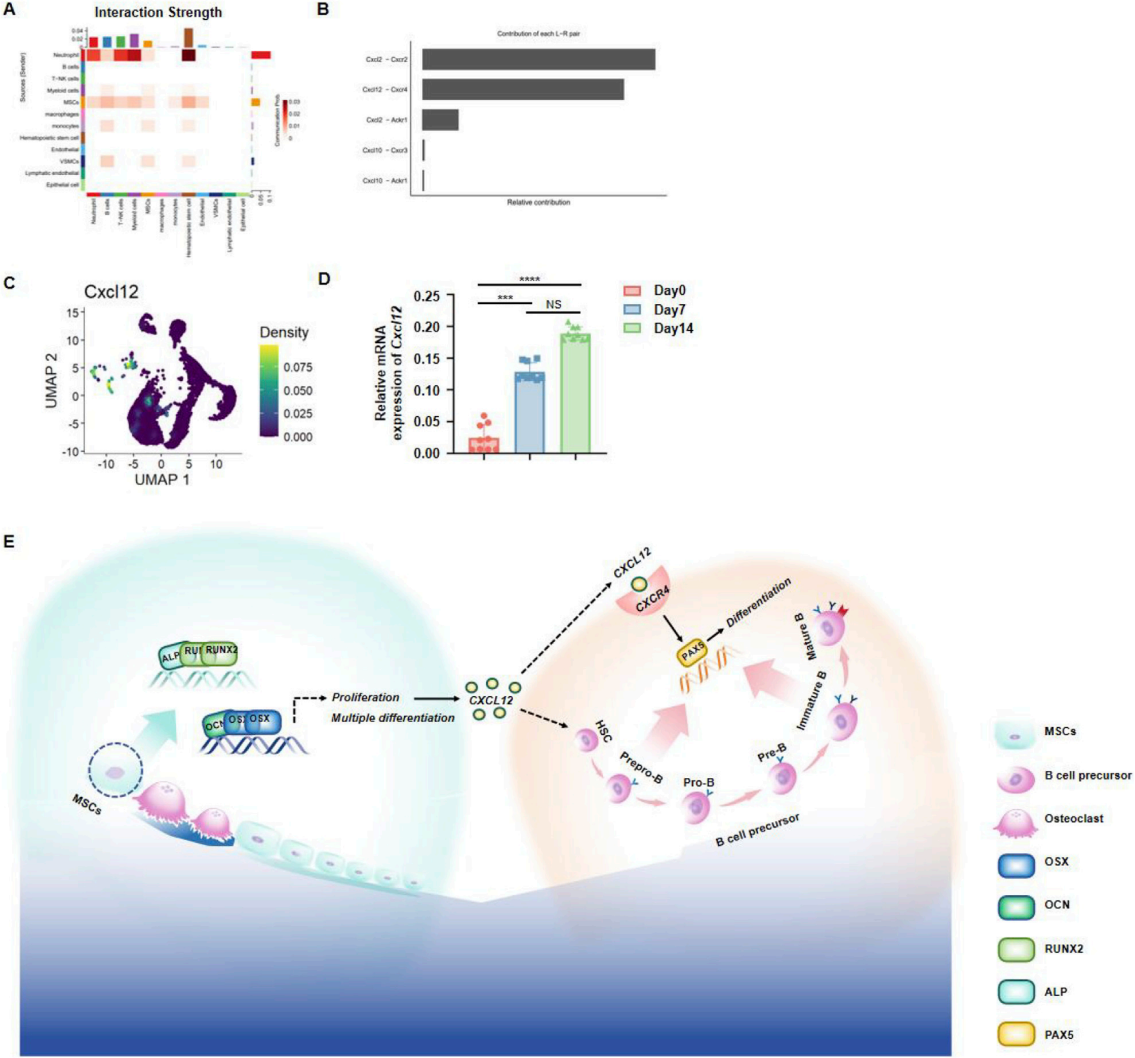
Jawbone mesenchymal stem cells regulate hematopoiesis through secretory function. **(A)** Heatmap of ligand–receptor interplay among various cell populations, where deeper hues indicate stronger interactions. **(B)** Bar chart displaying the relative contributions of prominent ligand–receptor pairs; CXCL2–CXCR2 and CXCL12–CXCR4 emerge as key axes of communication. **(C)** UMAP visualization of single cells, color‐coded by Cxcl12 expression levels. **(D)** qPCR results showing a time‐dependent increase in Cxcl12 mRNA during osteogenic differentiation (days 0, 7, and 14). **(E)** Schematic summarizing the mechanism by which jawbone MSCs secrete CXCL12 to support HSCs development, thereby modulating immune function.

To further investigate which cytokines secreted by jawbone MSCs govern the differentiation and development of hematopoietic stem cells, we performed an in‐depth reanalysis of our previously obtained single‐cell sequencing data of jawbone tissue. An intercellular interaction heatmap revealed strong associations between MSCs and hematopoietic stem cells (Figure 6A). By examining the relative contributions of paired ligand–receptor interactions, we found that the Cxcl2–Cxcr2 and Cxcl12–Cxcr4 axes exhibited notably higher contributions compared to other pairs, suggesting that these two pathways play a particularly significant role in the biological processes under study (Figure 6B). UMAP dimensional reduction further showed high CXCL12 expression within MSC subpopulations (Figure 6C). Consistent with these observations, qPCR analyses demonstrated a significant upregulation of *Cxcl12* during the osteogenic differentiation of jawbone MSCs (Figure 6D). In summary, we preliminarily demonstrated that jawbone MSCs may regulate the function of HSCs through CXCL12 (Figure 6E).

## 4 Discussion

Due to their distinct differentiation pathway and unique oral marrow microenvironment, jaw MSCs exhibit functionalities in bone repair and immune modulation that markedly differ from those derived from long bones. Studies indicate that jawbone‐derived bone marrow mesenchymal stem cells, in comparison with those from the femur, display superior osteogenic and angiogenic differentiation potential (Lin et al., 2021). B cells, which play a central role in immune regulation, antibody production, and adaptive immune responses, are highly dependent on their interactions with surrounding cells for development (Rastogi et al., 2022; Mauri, 2010). Osteoblasts, primarily known for their roles in bone formation and remodeling, have been shown to significantly modulate immune cell development through the secretion of cytokines and growth factors (Long, 2011; Wen et al., 2024). Within this framework, the interaction between osteoblasts and B lymphocytes in the jawbone has gained considerable attention in the field of osteoimmunology. Elucidating the molecular crosstalk between these cells within the jawbone microenvironment is critical for advancing our understanding of bone metabolism, periodontal disease progression, and bone regeneration. Furthermore, these insights offer new perspectives on how bone-derived signals may contribute to the regulation of local immune responses and broader systemic health.

In our study, we provide the first systematic description of the spatial relationship between osteoblasts and B cells within the jawbone of mice. Through histological analysis and immunofluorescence labeling, we observed a close physical proximity between these 2 cell types, suggesting a potential interaction within the local microenvironment. This spatial adjacency implies that osteoblasts may directly influence B cell behavior through cell-cell contact or paracrine signaling. Furthermore, our *in vitro* co-culture experiments and subsequent analytical techniques revealed a pivotal role of jaw osteoblasts in promoting B cell development and differentiation. The experimental data demonstrate that osteoblasts exhibit a significant capacity to support B cell maturation, a process mediated through the secretion of soluble factors. These findings underscore the importance of the jaw osteoblasts’ secretory functions in maintaining local immune homeostasis and suggest their broader role in the regulation of immune responses within the bone microenvironment.

Since our previous studies demonstrated that BMSCs significantly promote the differentiation of HSCs into B cells, we focused here on elucidating the mechanisms by which MSCs enhance B-cell development. To further clarify the molecular basis of jaw osteoblast-mediated B-cell regulation, we performed an in-depth analysis of their secretory factors and downstream signaling pathways. Single-cell RNA sequencing revealed that CXCL12 serves as the pivotal chemokine mediating the crosstalk between MSCs and B-cell differentiation. CXCL12, a chemokine predominantly secreted by MSCs, plays a pivotal role in immune and stem cell trafficking by binding to its receptor CXCR4. As a multifunctional bioactive factor, CXCL12 is critically involved in tissue repair and immune modulation (Sudo et al., 1993; Miller et al., 2002). Moreover, our molecular analyses demonstrated that cytokines secreted by jaw osteoblasts can activate the transcriptional activity of Pax5, a master regulator in B cell development. Pax5 is known to drive the differentiation of B cells from progenitor stages to mature states (Cobaleda et al., 2007). Therefore, by activating the Pax5 signaling pathway, jaw osteoblasts provide a supportive niche that enhances B cell maturation and functionality.

In this study, we employed an optimized enzymatic digestion approach to isolate and culture MSCs derived from the mandibles of adult C57 mice, and demonstrated their robust multilineage differentiation potential *in vitro*. Single‐cell sequencing revealed a strong interplay between jawbone MSCs and HSCs. Using multiplex tissue immunofluorescence, we observed that jawbone MSCs and HSCs were spatially adjacent within the mouse alveolar bone. Further development of a co‐culture system confirmed that these jawbone MSCs promote immune cell development and differentiation in a dose‐dependent manner. RT-qPCR analyses indicated that mandibular osteoblasts can transcriptionally activate various B cell–related genes, including Ebf1, Rag1, Il7r, and Pax5; notably, Pax5 functions as a pivotal transcription factor in B cell development. Immunofluorescence results showed that, under jawbone osteogenic induction, Pax5 expression was significantly elevated in B cells. An *in vivo* infection model further demonstrated that jawbone MSCs provide effective therapeutic benefits for infectious diseases.

## 5 Conclusion

Altogether, our findings suggest that murine jawbone MSCs secrete CXCL12 and other growth factors to establish a bone marrow microenvironment conducive to HSC survival and maturation, offering new insights into the immunomodulatory effects of stem cells and possible strategies for addressing infection‐related clinical challenges.

## Data Availability

The original contributions presented in the study are included in the article/Supplementary Material, further inquiries can be directed to the corresponding authors.
