# Supplementary material for: Jawbone mesenchymal stromal cells attenuate acute inflammation via hematopoietic niche reinforcement
Source: Front Bioeng Biotechnol. 2025 Aug 11;13:1596143. doi: 10.3389/fbioe.2025.1596143 (PMC12375904; doi:10.3389/fbioe.2025.1596143)
Supplement: Supplementary file 1 [file Supplementaryfile1.docx]

**Antibody list**

| **Antibodies** | **Host** | **Source** | **Catalog Number** | **Dilution** |
| --- | --- | --- | --- | --- |
| Anti-RUNX2 | Rabbit | Proteintech | 20700-1-AP | 1: 200 |
| Anti-Osteopontin | Rabbit | Proteintech | 22952-1-AP | 1: 200 |
| Anti-Sca-1 | Rat | Biolegend | 122501 | 1: 200 |
| Anti-PAX5 | Rabbit | [Cell Signaling Technology](https://www.cellsignal.cn/) | 12709T | 1: 200 |
| Anti-B220 | Rat | [Cell Signaling Technology](https://www.cellsignal.cn/) | 34399S | 1: 200 |

**Table S1. Primers for RT-qPCR**

| **Gene** | **Primer_F** | **Primer_R** |
| --- | --- | --- |
| ***βactin*** | ACCAACTGGGACGATATGGAGAAGA | TACGACCAGAGGCATACAGGGACAA |
| ***Runx2*** | CCAACCGAGTCATTTAAG | GCTCACGTCGCTCATCTTG |
| ***OSX*** | CCTTCCCTCACTCATTTCCTGG | TGTTGCCTGGACCTGGTGAGAT |
| ***ALP*** | CGGGACTGGTACTCGGATAA | ATTCCACGTCGGTTCTGTTC |
| ***OCN*** | TCTCTCTGCTCACTCTGCTGGCC | TTTGTCAGACTCAGGGCCGC |
| ***EBF1*** | GCATCCAACGGAGTGGAAG | GATTTCCGCAGGTTAGAAGGC |
| ***RAG1*** | ACCCGATGAAATTCAACACCC | CTGGAACTACTGGAGACTGTTCT |
| ***IL7R*** | GCGGACGATCACTCCTTCTG | AGCCCCACATATTTGAAATTCCA |
| ***PAX5*** | CCATCAGGACAGGACATGGAG | GGCAAGTTCCACTATCCTTTGG |
| ***Cxcl12*** | CCAGAGCCAACGTCAAGCAT | CAGCCGTGCAACAATCTGAA |
